# A134 THE PEDIATRIC ENDOSCOPY POCKET GUIDE – DEVELOPMENT OF A NOVEL “IN-THE-MOMENT” ELECTRONIC RESOURCE FOR PEDIATRIC ENDOSCOPY

**DOI:** 10.1093/jcag/gwac036.134

**Published:** 2023-03-07

**Authors:** R Sharma, L Hart, I Bayer, M Zachos

**Affiliations:** 1 Department of Pediatrics, Division of Gastroenterology and Nutrition; 2 Department of Pathology and Molecular Medicine; 3 Health Science Education Graduate Program, Faculty of Health Sciences, McMaster University , Hamilton, Canada

## Abstract

**Background:**

Endoscopy is vital to pediatric gastroenterology. Performing endoscopic procedures safely, effectively and with age-specific considerations requires integration of technical and cognitive competencies. There are many guidelines for endoscopic diagnosis, classification, and management of pediatric gastrointestinal conditions. To date, there are few collated resources for pediatric endoscopists.

Subspecialty trainee feedback at McMaster University identified the need for an accessible, consolidated resource to improve knowledge and competence. The literature shows a lack of exposure in subspeciality training to uncommon yet high acuity procedures such as gastrointestinal bleeding. In an apprenticeship-based learning model, this poses a challenge for both trainees and trainers. Evidence suggests use of multimedia tools is more effective for learning procedural skills than text alone, which guided the creation of this resource.

**Purpose:**

To develop an “in-the-moment” electronic resource to supplement training of pediatric endoscopic procedures.

**Method:**

A subspeciality trainee and two pediatric gastroenterologists with expertise in medical education and program development identified existing knowledge gaps. An electronic pocket guide with links to relevant guidelines and instructional resources was developed. The guide underwent six rounds of revision to ensure it contained relevant and updated guidelines. A copyright and e-learning expert reviewed the content to certify it complied with copyright laws and McMaster University’s accessibility guidelines. The guide will be uploaded to Pressbooks © which allows for export to multiple formats for distribution.

**Result(s):**

Based on knowledge gaps identified by local experts, the following six domains were incorporated into the pocket guide:

1. Logistics: safety procedures, colonoscopy preparation regimens, equipment specifications, considerations for urgent endoscopy and reporting guidelines

2. Troubleshooting: patient positioning, loop recognition/management strategies

3. Special tests: method of collection, collection media and lab protocols

4. Scoring systems: validated scoring tools for eosinophilic esophagitis, Barrett’s esophagus, esophageal varices, caustic esophageal injury, bleeding ulcers, and inflammatory bowel disease

5. Endoscopic emergencies: guidelines for esophageal burns, foreign body ingestions and upper gastrointestinal bleeds

6. Endoscopic tools: instructions for tools used in the categories of hemostasis, polypectomy, foreign body removal, esophageal varices, and strictures

**Conclusion(s):**

This novel teaching tool provides an electronic guide that can be used at all levels of pediatric gastroenterology training to gain familiarity and/or rapidly access resources for complex or infrequently encountered endoscopy techniques. Future studies will aim to evaluate the pocket guide from two aspects – ease of use/accessibility and whether implementation of this guide leads to increased competence with various endoscopic tools and techniques.

**Please acknowledge all funding agencies by checking the applicable boxes below:**

None

**Disclosure of Interest:**

None Declared

